# Steroid-Refractory Insulin Autoimmune Syndrome Treated With Rituximab and Continuous Glucose Monitoring

**DOI:** 10.7759/cureus.16513

**Published:** 2021-07-20

**Authors:** Chandar M Batra, Kiran Kumar, Monika Goyal

**Affiliations:** 1 Endocrinology, Indraprastha Apollo Hospitals, New Delhi, New Delhi, IND

**Keywords:** hyperinsulinemic hypoglycemia, systemic steroids, rituximab, spontaneous hypoglycemic attacks, insulin autoantibodies, very high insulin and c -peptide levels

## Abstract

A 67-year-old female presented with severe hypoglycemia with a blood glucose of 34 mg/dl five hours after having dinner. She did not have diabetes and had no access to oral hypoglycemic agents, insulin, or any other drug known to cause hypoglycemia. She was a known case of primary hypothyroidism euthyroid on treatment. The physical examination was unremarkable. Her liver, renal functions, thyroid, and adrenal functions were normal. At a blood sugar level of 23 mg/dl, her serum insulin was 24,000 uU/ml (normal: <3 uU/ml) and C-peptide was 16.2 ng/ml (normal: 0-0.6 ng/ml), which were were very high. As the serum insulin levels were very high, insulin autoimmune syndrome (IAS) was suspected. Insulin autoantibodies (IAAs) were positive [87.2 units/ml (normal: <12)]. Imaging with contrast-enhanced CT (CECT) of the abdomen, endoscopic ultrasonography, and 68 gallium octreotide DOTANOC whole-body PET-CT scan did not reveal any pancreatic or extra-pancreatic tumor. Eventually, the patient was diagnosed with IAS. She was started on high-dose prednisolone, diazoxide, and octreotide in addition to low carbohydrate meals. Hypoglycemic episodes continued for one month despite this therapy. Remission was achieved only after two doses of rituximab 1 g IV infusion were given. Serum insulin levels decreased to 230 uU units from 24,000 uU/ml, and the patient's hypoglycemic and hyperglycemic episodes were normalized. We used continuous glucose monitoring with the FreeStyle Libre glucose monitoring system, and the management of the patient was greatly facilitated by this.

## Introduction

Insulin autoimmune syndrome (IAS) was first described by Hirata in Japan in 1970 [1], and it is a rare disorder. Only 389 cases of IAS had been reported till 2009, mainly from Japan and China but very few in Caucasians and less than 10 from India [2,3]. IAS is characterized by severe spontaneous attacks of hyperinsulinemic hypoglycemia, high total immunoreactive insulin levels, elevated insulin autoantibody (IAA) titers, no prior exposure to exogenous insulin, and no pathological abnormalities of the pancreatic islet cells [4,5]. IAS is a rare disorder, and managing this case successfully gave us valuable insights into the pathogenesis of this disease at the molecular level.

## Case presentation

The patient was a 67-year-old woman who presented to the casualty of a local hospital at 3 am with complaints of severe anxiety, sweating, dryness of mouth, shortness of breath, and palpitation. Her medical history was remarkable for hypothyroidism and hypertension, and she was on amlodipine 5 mg daily and thyroxine 75 ug daily. The patient was not a known diabetic. There was no history of diabetes in family members, and the patient denied having access to any diabetic medication or any medication known to cause hypoglycemia. She had had her dinner at 9 pm and had been fairly asymptomatic prior to the presentation. On arrival to the ER, her pulse rate was 90/minute, BP was 170/100 mmHg, weight was 75 kg, and her BMI was 30 kg/m^2^. The physical exam was grossly unremarkable. She was conscious, oriented to time, place, and person, and had no apparent or gross neurological deficit. Her blood glucose in the ER was found to be 34 mg/dl. Immediate administration of dextrose led to the resolution of symptoms. However, the hypoglycemic attacks recurred the next day again, and she was put on a continuous 10% dextrose infusion. Despite the dextrose infusion, she had two to five episodes of nocturnal attacks of severe hypoglycemia with blood sugar levels of 30-40 mg/dl for the next 30 days. Blood counts, liver function tests, kidney function tests, and HbA1c were normal. Baseline ECG, chest X-ray, and ultrasound abdomen were also normal; serum cortisol levels were normal, and contrast-enhanced CT (CECT) of the abdomen revealed a normal pancreas and no extra-pancreatic tumor, but bilateral cortical scarring of both kidneys was present. Thyroid function tests were normal (Table 1).

**Table 1:** Investigations ANA: antinuclear antibodies; TPO: thyroid peroxidase; TSH: thyroid-stimulating hormone; CECT: contrast-enhanced computed tomography; PET: positron emission tomography

Variables	Results
At Diagnosis	
Blood glucose at the time of hypoglycemia	34 mg/dl
Insulin levels when blood sugar was 23 mg/dl	24,000 uU/ml
C-peptide level when blood sugar was 23 mg/dl	16.2 ng/ml
Insulin antibody	87.2 units/ml (normal: <12)
After Treatment	
Serum insulin levels	203 uU/ml
Insulin antibody	1.63 units/ml (normal: <12)
Imaging	CECT abdomen: negative; endoscopic ultrasonography: negative; 68 gallium DOTANOC whole-body PET scan: negative
Serum protein electrophoresis	Negative
Bone marrow aspiration and biopsy	Negative
Anti-dsDNA, ANA	Negative
Anti-TPO antibodies	33.1 u/ml (<60), negative
FT3	1.71 pg/dl (1.7-3.71)
FT4	1.00 ng/dl (0.7-1.48)
TSH	2.04 mIU/L (0.5-5)

A gallium octreotide DOTANOC whole-body PET-CT scan was done, with no abnormal uptake. The patient was referred to our hospital at this stage. Further investigations proved that she had hyperinsulinemic hypoglycemia. At a blood sugar level of 23 mg/dl, her serum insulin was 24,000 uu/ml (normal: <3 uu/ml) and C-peptide was 16.2 ng/ml (normal: <0.6 ng/ml), which were were very high. Autoimmune screen for RA factor, anti-CCP antibodies, ANA, and dsDNA antibodies were normal. Serum protein immunoelectrophoresis, Bence Jones proteins in urine, bone marrow aspiration, and biopsy were normal, ruling out multiple myeloma. A diagnosis of IAS was made, and the patient was started on a 1,600 kcals low carbohydrate diet divided into six meals a day. Prednisolone 60 mg daily was started along with diazoxide 50 mg thrice daily, octreotide 50 ug subcutaneous eight hourly before meals, and thiazide diuretic 50 mg twice daily. The frequency of hypoglycemic episodes decreased, but the patient still had at least one or two severe hypoglycemic episodes a day. Monitoring of sugar levels was done every two hours and SOS using continuous glucose monitoring with the FreeStyle Libre glucose monitoring system.

**Figure 1:**
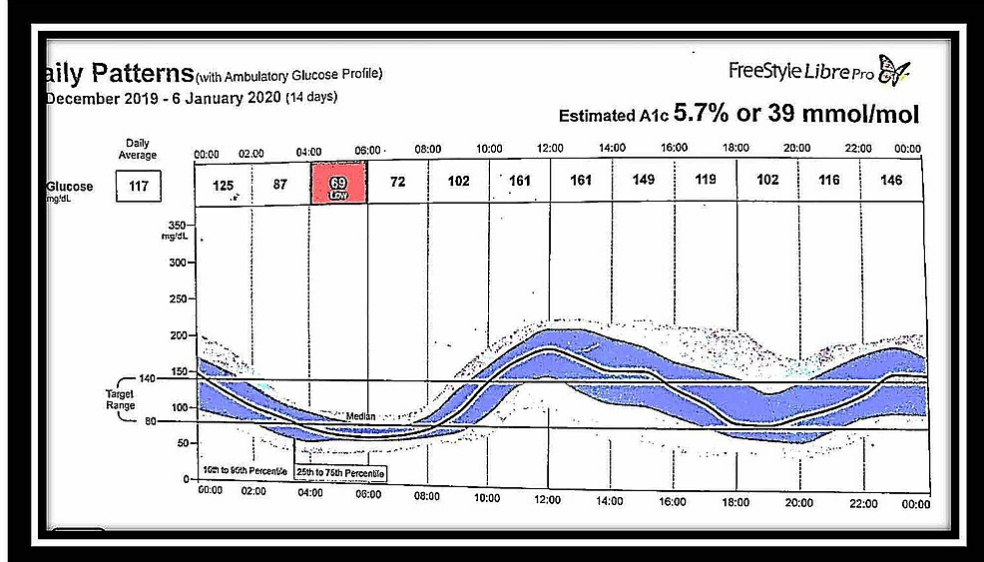
FreeStyle Libre glucose monitoring system readings: December 24, 2019-January 6, 2020 – chart 1 The chart shows daily hypoglycemic attacks between 4-8 am and hyperglycemic episodes between 12 noon and 4 pm during the period from December 24, 2019, to January 6, 2020, after 40 days of steroid therapy

**Figure 2:**
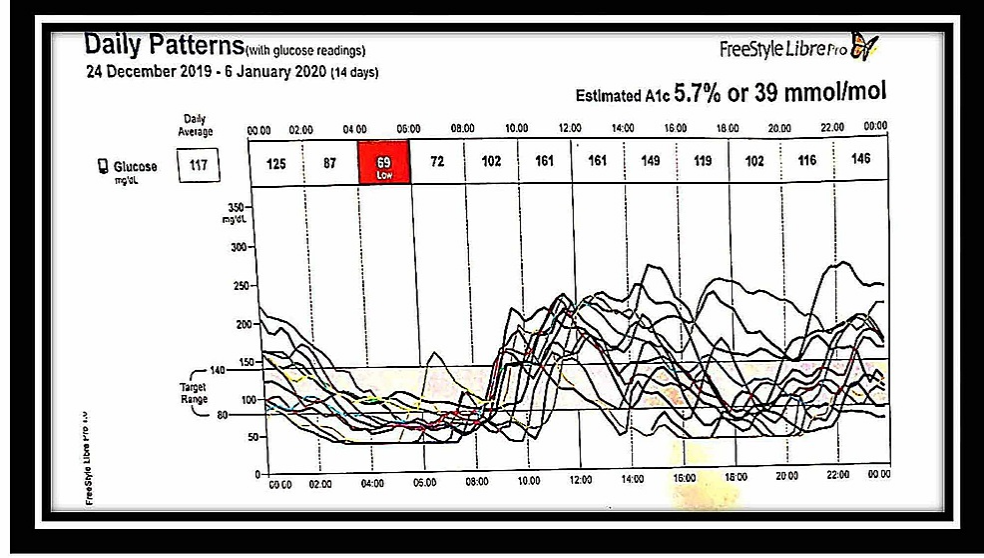
FreeStyle Libre glucose monitoring system readings: December 24, 2019-January 6, 2020 – chart 2 The chart shows multiple episodes of severe hypoglycemia from December 24, 2019, to January 6, 2020, after one month of steroid therapy. The chart also shows multiple episodes of hyperglycemia

The patient was maintained on this regime for three more weeks. However, the frequency of severe hypoglycemic episodes was one per day, and the duration of blood sugar levels below 60 mg/dl was four hours a day, and that of blood sugars above 200 mg/dl going to a maximum of 300 mg/dl was about six hours/day, along with 14 hours of euglycemia (Figures 1, 2).

We added metformin sustained release 500 mg twice daily to decrease the hyperglycemic episodes. The patient was given an injection of rituximab, a monoclonal antibody acting against CD20 helper cells to decrease insulin antibodies. The hypoglycemic episodes decreased within seven days to one episode of severe hypoglycemia every three days, and the patient was discharged on the FreeStyle Libre glucose monitoring system. On day 53, the patient was admitted for complaints of severe giddiness, nausea and vomiting, and body ache for two days. Examination revealed mild fluid overload in the form of pedal edema and basal crepitations and pedal edema. Labs showed severe hypervolemic hyponatremia with serum sodium of 108 meq/l, urine sodium of 10 meq/l. Hyponatremia was successfully treated with 3% normal saline followed by five days of tolvaptan. Diazoxide and hydrochlorothiazide were discontinued.

**Figure 3:**
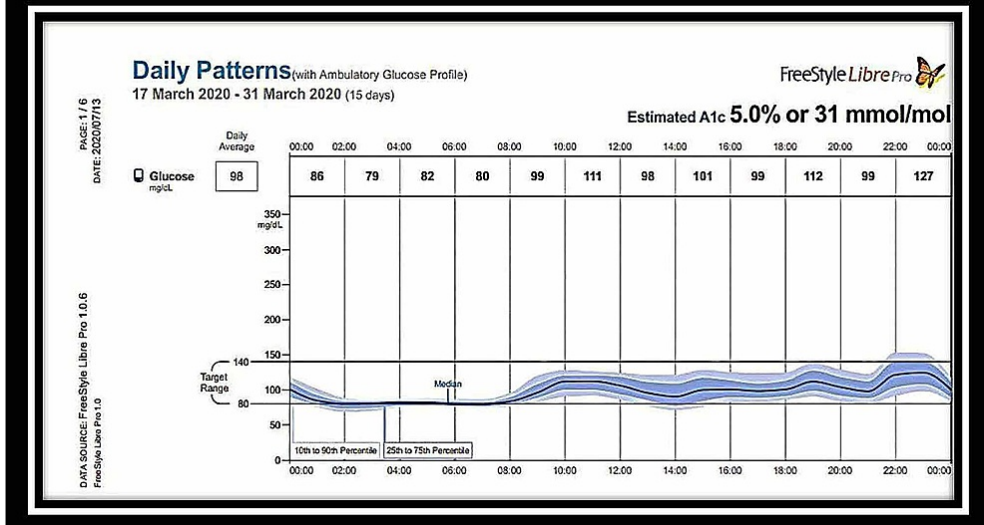
FreeStyle Libre glucose monitoring system readings: March 17-31, 2020 – chart 1 The chart readings from March 17-31, 2020, after one month of two doses of rituximab, showing a euglycemic pattern

**Figure 4:**
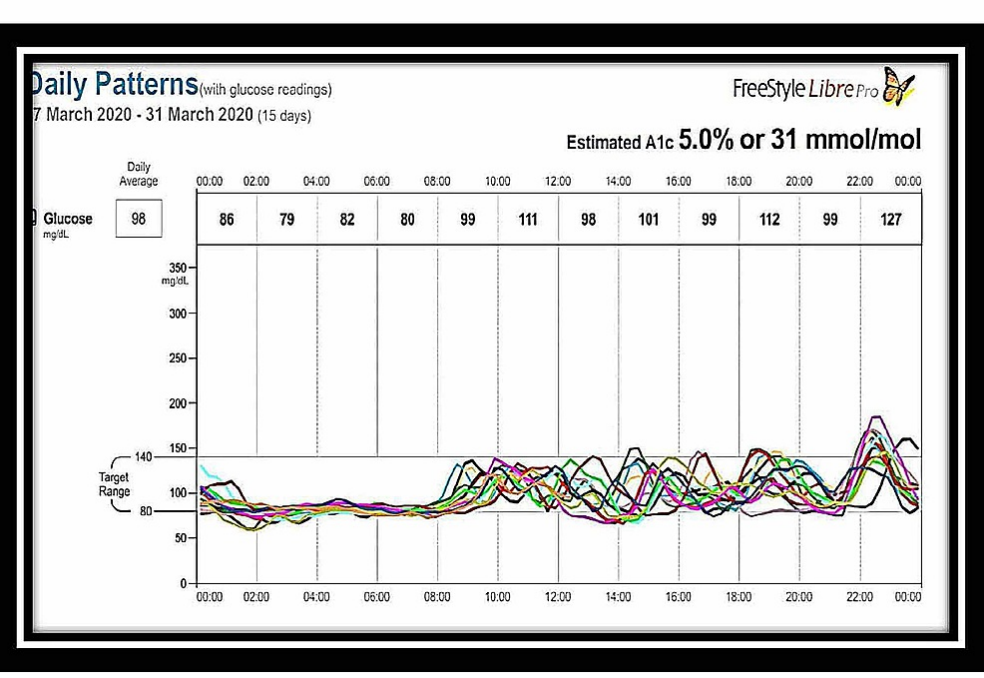
FreeStyle Libre glucose monitoring system readings: March 17-31, 2020 – chart 2 The chart readings from March 17-31, 2020 showing normalization of hypoglycemia and hyperglycemia after two doses of rituximab therapy

A second dose of rituximab 1 g IV infusion was given. The insulin antibodies became normal, and serum insulin levels also decreased from 24,000 uU/ml to 200 uU/ml after three months of the second dose of rituximab (Table 1). The patient was monitored at home with a FreeStyle Libre continuous glucose monitoring system, which showed no hypoglycemic episodes after one month of the second dose. However, the blood glucose was in the lower normal range (Figures 3, 4).

Differential diagnosis

A complete history, physical examination, and investigations were done to rule out any drug ingestion that could have caused hypoglycemia as well as to rule out critical illnesses like renal, liver, and cardiac failure, and adrenocortical deficiency. The patient had experienced significant episodes of spontaneous hypoglycemia, which were symptomatic with blood sugars levels of 23-60 mg/dl relieved with glucose ingestion, thus satisfying all of Whipple's triad criteria. She also had hyperinsulinemic hypoglycemia. The values that confirm hyperinsulinemic hypoglycemia are serum insulin >3 uU/ml and C-peptide >0.6 ng/ml [6]. Our patient had serum insulin of 2,400 uU/ml at a blood sugar level of 23 mg/dl, which clinched the diagnosis in favor of IAS due to insulin antibodies. Exogenous insulin administration was ruled out due to increased levels of both serum insulin and C-peptide. We could not do a screening for sulphonylurea drugs as it is not available in our country. However, no one in the patient's family was diabetic, and the patient had no access to insulin or oral hypoglycemic agents. Autoimmune screen for RA factor, anti-CCP antibodies, ANA, dsDNA antibodies, as well as anti-TPO antibodies were normal. Serum protein immunoelectrophoresis, Bence Jones proteins in urine, bone marrow aspiration, and biopsy were normal, ruling out multiple myeloma. Insulin receptor antibodies were not tested because the patient did not have insulin resistance features, acanthosis nigricans, hyperandrogenism, and severe resistant hyperglycemia requiring huge amounts of insulin, alternating with hypoglycemia. The final diagnosis was IAS, but no precipitating factor was found.

Treatment

The patient was started on both diet and drug therapy. Diet therapy included a frequent low carbohydrate diet divided into six meals per day. Pharmacological management included prednisolone 60 mg once a day, octreotide 50 mcg, diazoxide 50 mg three times a day after meals, and hydrochlorothiazide 50 mg daily. However, the hypoglycaemic episodes did not subside even after one month of this therapy. The patient was considered steroid-resistant as the hypoglycemic episodes were recurring even after initiating steroid therapy (Figures 1, 2). Despite the supraphysiological high-dose steroids, the requirement of continuous glucose infusion and signs of steroid toxicity prompted us to look for other therapeutic options. The patient was offered rituximab therapy. The first dose of rituximab 1 g IV infusion was given along with methylprednisolone 125 mg intravenously as premedication. One hypoglycemic episode was observed in the next two weeks. The patient developed severe hyponatremia after 10 days, which was successfully treated with 3% normal saline and tolvaptan. Diazoxide and hydrochlorothiazide were stopped. A subsequent second dose of rituximab 1 g IV infusion was given. The patient was monitored at home with the FreeStyle Libre continuous glucose monitoring system, which showed no further hypoglycemic episodes. However, the blood sugar was in the lower normal range, as shown in Figures 3, 4. She had hypertension on admission and was on amlodipine 5 mg daily.

Outcome and follow-up

Prednisolone was tapered over the next three months. No hypoglycemic episodes were noted clinically in the follow-up period. The patient developed a persistent backache, for which we evaluated her with a DEXA scan for the hip and spine, which revealed osteoporosis. Zoledronic acid 5 mg was given for this postmenopausal and steroid-induced osteoporosis, which led to improvement of her back pain. The patient had also gained weight, developed proximal myopathy, and facial puffiness due to steroid therapy. She was monitored with the FreeStyle Libre continuous glucose monitoring system after completing the therapy, which showed complete cessation of hypoglycemic and hyperglycemic episodes (Figures 3,4). The patient's fasting serum insulin levels, which had been >24,000 uU/ml earlier, reduced to 235 uU/ml at the end of three months post-therapy, and her insulin antibody levels, which had been positive, became negative.

## Discussion

IAS is a rare autoimmune syndrome first described by Hirata et al. in 1970 [1]. It is characterized by severe attacks of spontaneous hyperinsulinemic hypoglycemia associated with elevated serum immunoreactive insulin levels, increased insulin autoantibody titers, no prior exposure to exogenous insulin, and normal pancreatic beta cells [2,3]. Insulin autoantibodies are usually of immunoglobulin G (IgG) class and polyclonal but can be monoclonal and of IgA or IgM class as well. The insulin secreted after a meal is bound by these autoantibodies forming insulin-insulin antibody complexes. This causes hyperglycemia due to decreased free insulin, resulting in more insulin secreted by the beta cells. When the antibodies saturate their capacity to absorb insulin, they suddenly dissociate, releasing a large amount of free insulin into the bloodstream. This sudden release of insulin, which does not correlate with food intake or blood sugar, causes hypoglycemic attacks. The hypoglycemic episodes can occur at any time of the day in the post-absorptive, postprandial, or fasting state [2]. The severity of the hypoglycemic attacks is increased because hyperinsulinemia inhibits glycogenolysis and gluconeogenesis, and the formation of ketone bodies, thereby depriving the body of the use of an alternative fuel source [7]. Recurrent hypoglycemic attacks decrease the counter-regulatory hormone response to hypoglycemia, as a result of which the secretion of cortisol, catecholamines, and glucagon is impaired in response to hypoglycemia. This decreases the threshold of the patient for the perception of hypoglycemia. The pathogenesis of this disease is probably secondary to genetic predisposition and environmental factors. Drugs and viral infections could also act as triggers (Table 2).

HLADR4 is the most susceptible gene, mainly DRB10406, DRB10403, and DRB10407. The list of drugs implicated has either a sulphydryl (SH) group or their metabolites contain the SH group. The SH group cleaves the insulin molecule's disulfide bond, leaving the linear fragment on the α-chain exposed to the HLADR4 on antigen-presenting cells that bind it. This activates specific T helper cells, which activate the B lymphocytes to produce IAAs [2]. Autoimmunity to insulin is triggered by many factors, including viral infections and exposure to certain medications (Table 2). In our patient, no trigger could be found. Patients of Asian origin have been described, where a trigger is not found [2,4]. This syndrome's clinical picture is variable, ranging from mild hypoglycemic attacks to hypoglycemic coma and death due to hypoglycemia. This alternates with hyperglycemic periods when blood sugars can go up to 500 mg/dl. Our patient had severe hypoglycemic attacks requiring IV glucose and hospitalization, and there were daily periods of hyperglycemia. These hyperglycemia periods were due to decreased free insulin levels due to the insulin-insulin antibody complex formation. There was also a component of steroid-induced hyperglycemia as the patient was on 60 mg of prednisolone. The American Endocrine Society guidelines state that a Whipple's triad has to be seen [6]. There were multiple episodes of symptomatic hypoglycemia in our patient in which blood sugar levels as low as 23 mg/dl were recorded, and these hypoglycemic symptoms were reversed with glucose administration. The diagnosis of hyperinsulinemic hypoglycemia also satisfied the American Endocrine Society's criteria that at the time of hypoglycemia, when blood sugar is less than 55 mg/dl, the serum insulin should be >3 uU/dl and C-peptide should be >0.6 ng/dl [6]. At a blood sugar level of 23 mg/dl, the patient's serum insulin was 2,400 uU/dl. Other systemic autoimmune disorders are usually found in patients who have IAS. Our patient did have primary hypothyroidism, but anti-TPO antibodies were negative, cortisols were within normal limits, ANA and anti-ds-DNA antibodies were negative, and there was no other evidence of the autoimmune disorder. IAS also has an association with multiple myeloma and monoclonal gammopathy [2], but serum protein electrophoresis and bone marrow biopsy were normal in our case. IAS is a self-limiting disorder once the triggering factor is withdrawn in three to six months. In our case, no triggering factor was found.

**Table 2:** Factors triggering IAS* *[2] IAS: insulin autoimmune syndrome

Drugs	Drugs	Viruses
Methimazole, propyluracil	D penicillamine	Chickenpox
Alpha-lipoic acid	Hydralazine	Mumps
Gliclazide	Clopidogrel	Rubella
Captopril	Pantoprazole	Hepatitis C
Penicillin G	Methionine	Measles
D penicillamine	Glutathione	
Imipenem	Mercaptans	
Isoniazid		

No definite guidelines are currently available for the management of IAS. Most patients with IAS manifest mild hypoglycemic episodes, which are easily treated with diet therapy. Usually, treatment is initiated with diet therapy consisting of a low carbohydrate diet divided into six meals per day. Deguchi et al. have described the effectiveness of diet therapy for hypoglycemia. They found good results with cornstarch for improving hypoglycemic symptoms [8]. Decreasing carbohydrate intake works by decreasing insulin production. Decreasing the absorption of carbohydrates by acarbose works the same way. We started our patient on a complex carbohydrate diet and drug therapy as she had been having life-threatening hypoglycemic episodes. The next therapy line is to decrease insulin production from the beta cells directly by diazoxide or octreotide. We also started diazoxide and octreotide, but the patient continued to have severe hypoglycemic episodes. On the fifth day after admission, the patient was started on high-dose prednisolone 1 mg/kg/day. Prednisolone in high doses is used for one to six months in many studies and has been found to relieve hypoglycemic attacks and decrease insulin antibody titers effectively [2,4]. Our patient was still hypoglycemic despite one month of steroid therapy and was developing signs of steroid toxicity. The treatment options for steroid-refractory IAS are azathioprine, rituximab, and plasmapheresis [2-4]. Rituximab was tried in IAS based on the results of the trialNET study, which was conducted for type 1 diabetes patients. In this study, rituximab was able to reduce titers of anti-insulin antibodies [9]. Rituximab is an anti-CD20 monoclonal antibody that is used to treat severe refractory IAS both alone [2,4,10,11] and, following immunoadsorption, with an adsorber system containing sheep antigens directed against human immunoglobulins [12]. In our patient, post-rituximab therapy, antibody titers were reduced to normal, and insulin levels also decreased significantly. The patient was free of hypoglycemic and hyperglycemic episodes and was in remission, as is evident in the studies with continuous glucose monitoring with the FreeStyle Libre glucose monitoring system (Figures 3, 4). One other option available was plasmapheresis [13], which we did not use. Continuous glucose monitoring with the FreeStyle Libre glucose monitoring system is an excellent tool in managing IAS. Real-time blood sugar values can be obtained from the sensor with the help of a scanner. It enables the reduction in the number of pricks to the patient. It also gives the value of total time in hypoglycemia and total time in the range (TIR). TIR and time in hypoglycemia guide the physician in selecting an appropriate method for managing IAS [2]. Saxon et al. have achieved similar results with real-time continuous monitoring of glucose system (RT-CMGS) [10].

## Conclusions

IAS presents with a spectrum of hypoglycemic attacks varying from mild hypoglycemic attacks to severe hypoglycemic attacks leading to convulsions and coma. The hypoglycemic attacks can occur at any time in the postprandial or fasting state. Very high insulin and C-peptide levels and positive insulin antibodies in a patient with hyperinsulinemic hypoglycaemic attacks are diagnostic of this disease. High-dose corticosteroids should be started if the patient is not responsive to diet therapy. Steroid resistance is suspected if hypoglycemic episodes do not subside with supraphysiological doses of corticosteroids, and then the patient is treated with immunosuppressants. Rituximab, azathioprine, and plasmapheresis have all been used successfully in these resistant cases, but the best results have been achieved with rituximab. Continuous glucose monitoring using the FreeStyle Libre glucose monitoring system is a valuable tool for monitoring treatment efficacy and cure in IAS patients.
